# Invasive Group B *Streptococcus* Infections Caused by Hypervirulent Clone of *S. agalactiae* Sequence Type 283, Hong Kong, China, 2021^1^

**DOI:** 10.3201/eid3101.231627

**Published:** 2025-01

**Authors:** Carmen Li, Herman Tse, Chendi Zhu, Garnet Kwan Yue Choi, Alfred Lok-Hang Lee, Jun Yang, Norman Wai-Sing Lo, Daisy Tsz-Yung Hui, Christina Kin-Yi Chow, Sandy Ka-Yee Chau, Jimmy Lam, Kristine Luk, Tak-Lun Que, Kitty Sau-Chun Fung, Cindy Tse, Sally Cheuk-Ying Wong, David Christopher Lung, Viola Chi-Ying Chow, Margaret Ip

**Affiliations:** The Chinese University of Hong Kong, Hong Kong Special Administrative Region of China (C. Li, C. Zhu, J. Yang, N.W.-S. Lo, M. Ip); Khoo Teck Puat Hospital, Singapore (H. Tse); Hong Kong Children’s Hospital, Hong Kong (G.K.Y. Choi, S.C.-Y. Wong, D.C. Lung); Prince of Wales Hospital, Hong Kong (A.L.-H. Lee, V.C.-Y. Chow); Queen Elizabeth Hospital, Hong Kong (D.T.-Y. Hui, C.K.-Y. Chow, S.C.-Y. Wong, D.C. Lung); United Christian Hospital, Hong Kong (S.K.-Y Chau, K.S.-C. Fung); Pamela Youde Nethersole Eastern Hospital, Hong Kong (J. Lam); Princess Margaret Hospital, Hong Kong (K. Luk); Tuen Mun Hospital, Hong Kong (T.-L. Que); Kwong Wah Hospital, Hong Kong (C. Tse)

**Keywords:** *Streptococcus agalactiae*, group B Streptococcus, GBS, ST283, meningitis, sepsis, bacteremia, pyogenic arthritis, molecular epidemiology, streptococcal infections, Hong Kong, China, streptococci

## Abstract

During September–October 2021, group B *Streptococcus* bloodstream infections surged among patients hospitalized in Hong Kong. Of 95 cases, 57 were caused by the hypervirulent strain sequence type 283, which at the time was also found in freshwater fish and wet market environments and thus poses a transmission threat.

In 2015, the zoonotic potential of group B *Streptococcus* (GBS) sequence type (ST) 283 was highlighted in the Singapore outbreak of bacteremia cases associated with consumption of raw freshwater fish, which led to the ban of raw freshwater fish in all ready-to-eat raw fish dishes in Singapore (*1*,*2*). ST283 was found not only among patients with GBS bacteremia in Southeast Asia but also associated with aquaculture (*2*–*4*). ST283 was first noted to cause infection in humans in the mid-1990s, and its invasiveness was described in meningitis and bacteremia cases in 2000 and 2006, respectively (*5**,**6*). Since then, outbreaks among humans in Singapore and among freshwater fish species in Southeast Asia and Brazil have been noted (*3*,*4*,*7*,*8*). During September–October 2021, a surge of ST283 invasive GBS (iGBS) disease among nonpregnant adults was reported in public hospitals in Hong Kong, China, in response to which the Centre for Health Protection (CHP) issued a special bulletin on the investigation and heightened surveillance of the group B Streptococcus invasive disease (*9*). Because consumption of raw freshwater fish is prohibited in Hong Kong, other potential sources or transmission routes of the strain were investigated. We report the molecular epidemiology of GBS ST283 and the clinical characteristics of infections during that period.

## The Study

During September 2–November 6 (weeks 35–44) of 2021, a total of 95 cases of iGBS infections were reported from 17 public hospitals across Hong Kong. GBS isolates were characterized by whole-genome sequencing. In addition, 11 GBS strains were isolated from fish and environmental samples collected from wet markets at week 39. Clinical and laboratory data retrieval were approved by the Central Institutional Review Board of the Hospital Authority (reference no. CIRB2022-056-5) and the Joint New Territories East Cluster–Chinese University of Hong Kong Ethics Committee (reference no. CREC2018.509).

We confirmed the identity of GBS isolates by using matrix-assisted laser desorption/ionization time-of-flight mass spectrometry (Bruker Daltonics, https://www.bruker.com) and extracted DNA by using the QIAGEN EZ1 Virus Mini Kit v2.0 (QIAGEN, https://www.qiagen.com) on the EZ1 Advanced XL platform according to the manufacturer’s protocol. We prepared libraries by using the Illumina Nextera XT DNA Library Preparation Kit (Illumina, https://www.illumina.com) according to instructions and performed sequencing by using an Illumina sequencer with an average of 60× coverage. The pipeline of genome assembly and matching of STs, antimicrobial resistance genes, and virulence factors have been previously described (*10*). We mapped assembled genomes to reference genome SG-M158 (GenBank accession no. CP021864) by using Snippy v4.6.0 (http://github.com). For comparison, we included archived ST283 genomes CU_GBS_98, CU_GBS_08 from Hong Kong and SG-M1 from Singapore (GenBank accession nos. CP010875.1, CP010874.1, and CP012419.2). Variants were called by Freebayes v1.3.6 (https://github.com/freebayes/freebayes), and sites of single-nucleotide polymorphisms (SNPs) were further analyzed. We identified recombination sites and filtered them by using Gubbins v3.1.0 (https://github.com/nickjcroucher.gubbins) and generated a whole-genome SNP tree by using IQ-TREE v2.2.0.3 (https://github.com/iqtree/iqtree2) and autoselected model (TVMe+ASC+R2). Branch support was provided by UFBoot with >1,000 iterations. Sequence data of the strains are available under National Center for Biotechnology Information BioProject no. PRJNA999453.

**Figure 1:**
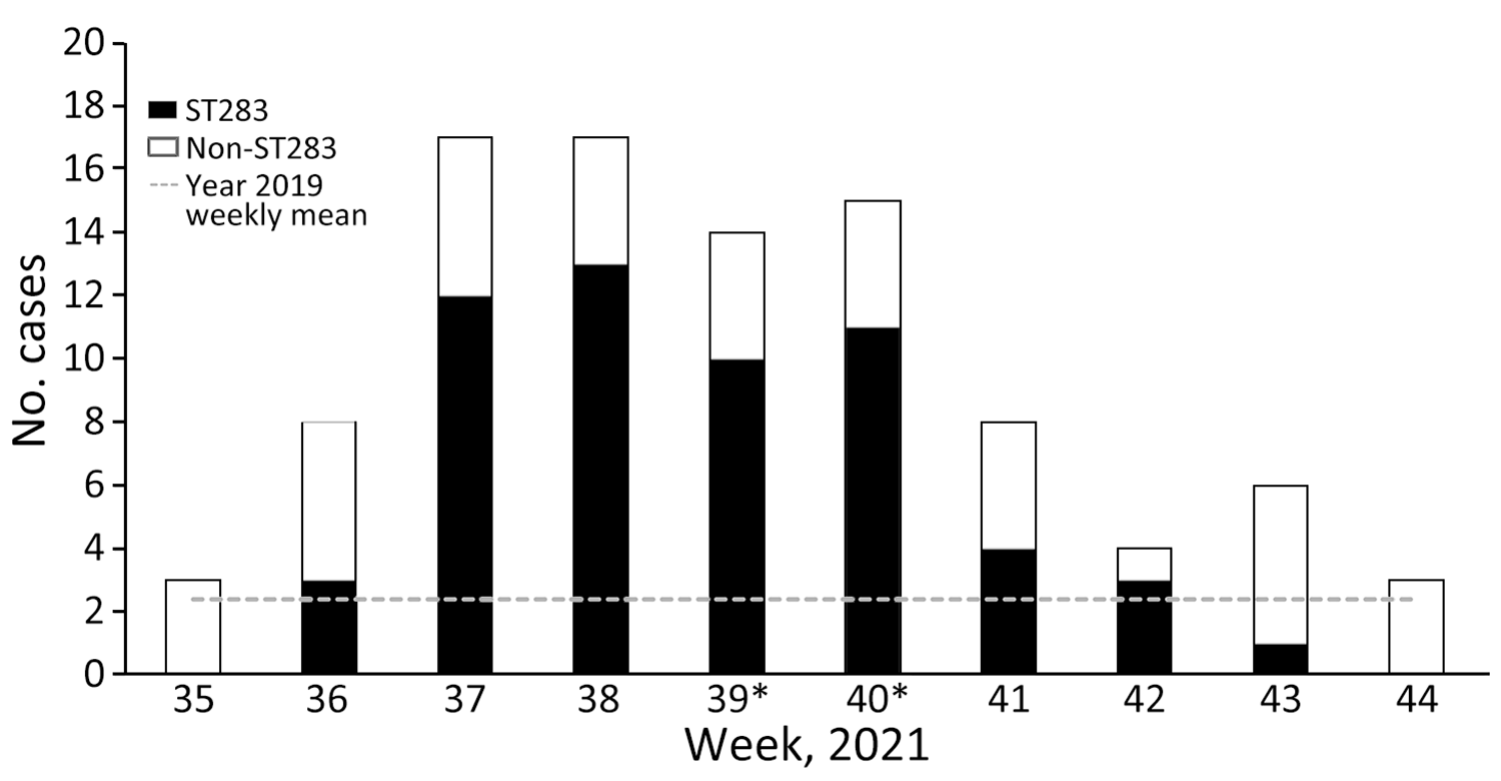
Invasive group B *Streptococcus* (GBS) infection incidence from September 2–November 6 (weeks 35–44), 2021, in 17 public hospitals across Hong Kong, China. The gray dotted line indicates the weekly mean number of invasive GBS infections for the same weeks in 2019. *Environmental sampling for GBS occurred during week 39 at local wet markets, and during week 40, a special bulletin was issued to raise public awareness of the upsurging cases. ST, sequence type.

The number of GBS bacteremia cases surged during weeks 37–40, when 14–17 cases per week were reported (Figure 1), which was 4-fold higher than baseline in 2019. During weeks 36–43, a total of 57 (60%) cases belonged to ST283, and the last case of ST283 infection was observed in week 43. The mean age for the overall iGBS cohort was 66.7 ± 17.8 years (range 1 month–96 years), and ST283 cases were limited to nonpregnant adults (age range 31–90 years, mean 66.2 ± 12 years) (Table). The male:female ratio was the same for patients in non-ST283 and ST283 cohorts, and mortality rates were 7.9% (3/38) for patients with non-ST283 infection and 8.8% (5/57) for patients with ST283 infection. Joint infections with involvement of single or multiple joints was common in patients with ST283 infection (26.3%) (p = 0.02). We found no statistically significant difference in the number of comorbidities between cohorts with ST283 and those with non-ST283 infections. During the study period, other STs found in patients with iGBS infection were ST1, ST17, ST890, and ST12. Among the 11 nonhuman GBS isolates, 3 fish strains belonged to serotype Ia ST7, and 8 were ST283. Both ST7 and 283 have been associated with disease in fish (*4*,*5*,*8*,*10*), suggesting that they were present in food animals before harvest rather than contaminated after harvest. 

**Table:** Characteristics of patients with group B *Streptococcus* infection during upsurge of cases in Hong Kong, September 2–November 6 (weeks 35–44), 2021*

Characteristic	Total, n = 95	Non-ST283, n = 38	ST283, n = 57	p value†
Age, y				
Mean ± SD	66.7 ± 17.8	67.2 ± 24.3	66.4 ± 12	0.7
Range	0–96	0–96	31–90	
Age group				
<65	39	13	26	0.19
>65	56	25	31	
Sex				
M	50	20	30	0.58
F	45	18	27	
Length of hospital stay, d				
Average	24.4	25.7	23.6	0.76
Range	1–202	1–202	1–100	
Median (IQR) C-reactive protein, mg/dL	NA	7.2 (2.7–11.9)	18.3 (11.6–24.9)	**<0.001**
Median (IQR) Urea, mmol/L	NA	7.45 (4.9–11.3)	6.7 (4.8–9.1)	**0.03**
Death outcome during admission	8	3	5	1
Specimen				
Blood	93	38	55	0.5
Other‡	2	0	2	NA
Clinical diagnoses/symptoms§				
Septicemia/sepsis	43	18	26	0.52
Joint infection	18	3	15	**0.02**
Skin infection	10	6	4	0.31
Meningitis	7	1	6	0.23
Urinary tract infection	7	5	2	0.31
Pneumonia	6	4	2	0.21
Comorbidities§				
Diabetes mellitus	11	5	6	0.75
History of any tumor/ cancer	7	5	2	0.11
Congestive heart failure	6	2	4	1
Myocardial infarction	3	1	2	1
Cerebrovascular disease	4	1	3	0.64
Moderate to severe renal disease	3	3	0	0.06
Peripheral vascular disease	2	0	2	0.51
Dementia	2	2	0	0.15
Moderate to severe liver disease	2	0	2	0.51
Comorbidity score >2	5	3	2	0.64
Hospital cluster of cases				
Hong Kong East	11	3	8	0.52
Kowloon Central	21	10	11	0.46
Kowloon East	17	6	11	0.79
Kowloon West	21	8	13	1
New Territories East	12	4	8	0.76
New Territories West	13	7	6	0.36
ST				
283	57	NA	57	NA
1	11	11	NA	NA
17	4	4	NA	NA
890	4	4	NA	NA
12	3	3	NA	NA
Other¶	16	16	NA	NA

*Values are no. patients except as indicated. Boldface indicate significance. NA, not applicable; IQR, interquartile range; ST, sequence type. †p values were calculated by χ^2^ test or *t*-test where appropriate. ‡Includes cerebrospinal fluid, joint fluid, and tissue. §A patient can have multiple symptoms and comorbidities. ¶STs isolated from <2 patients, including ST3, ST7, ST10, ST19, ST28, and ST654 (2 isolates/ST) and 1 each of ST24, ST27, ST452, and ST739.

**Figure 2:**
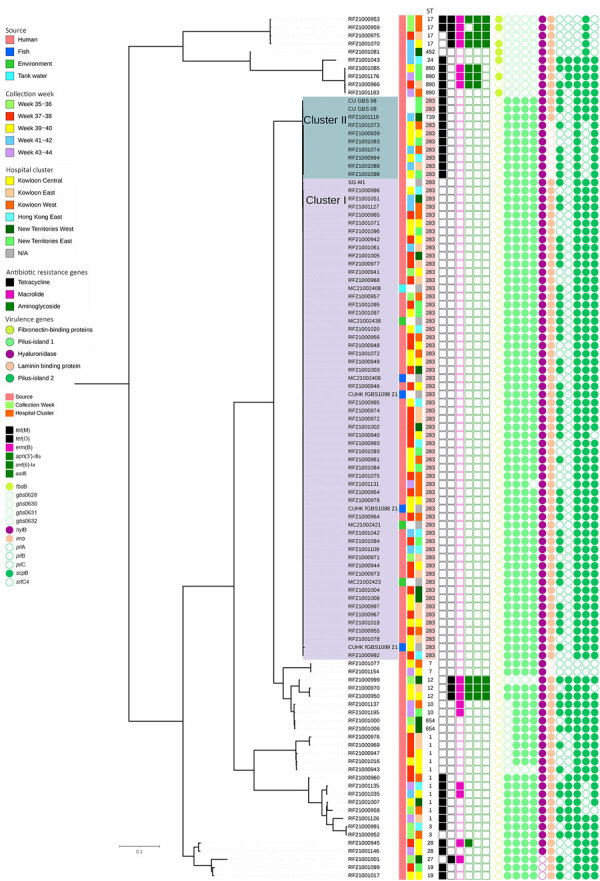
Whole-genome single-nucleotide polymorphism tree of group B *Streptococcus* isolates from patients, freshwater fish, and wet market environment in Hong Kong, China**.** The tree was rooted at midpoint. Demographics (including the week of isolate collection, hospital cluster, and source of isolate) and molecular characteristics (including STs, presence of antimicrobial resistance genes and virulence genes) of the isolates are indicated in the legend on the left side of the figure. ST283 highlighted in pink under column ST. Visualization of the tree was performed by using iTOL (https://itol.embl.de). ST, sequence type.

Antibiotic susceptibility testing following Clinical Laboratory and Standards Institute guidelines indicated that all GBS strains were sensitive to penicillin (*11*). Genome analysis showed that the ST283 isolates had 0–1037 SNPs with an average distance of 484 SNPs. Two clades of ST283 were depicted by the presence of the *TetM* gene (Figure 2, panel A). The main clade (cluster I), which consisted of 57 isolates (including 3 from fish, 4 from wet market environment, and 1 from tank water), had no antimicrobial resistant genes and clustered with SG-M1. Among the 57 isolates, 33 (67%) of 49 were from patients who had a history of handling raw fish, and that cluster led to the upsurge of cases in hospitals. A minor clade of 7 ST283 strains (cluster II) carried the *tetM* gene on Tn916 and clustered with archived genomes (CU_GBS_98 and CU_GBS_08) along with a ST739 strain (a single-locus variant of ST283 at the *adhP* gene). Compared with the archived genomes and ST739, those strains also lacked *lmb* and *scpB* genes. *Lmb* encodes for laminin-binding protein and *scpB* for part of the pilus island for invasion to host epithelial cells. We compared the 2 clusters with 303 ST283 genomes from the National Center for Biotechnology Information (Appendix 1). Cluster I was a separate clade from the Singapore outbreak and showed convergence to strains from Thailand (Appendix 2). Cluster II was also observed in species of fish in Southeast Asia (*4*,*5*,*8*).

According to the Hong Kong Observatory, the mean ambient temperatures were 29.7°C in September and 26°C in October 2021**.** September was one of the hottest months of the year, which concurred with previous findings of higher prevalence rates of GBS isolation from food animals and iGBS disease caused by ST283 in patients during the summer, resulting from the high mean temperature (>28°C) (*10*,*12*). CHP issued a special bulletin with regard to the ST283 upsurge, when a history of handling raw fish was noted. Consumption of raw fish from dining outlets could be ruled out because selling raw freshwater fish sashimi had been prohibited in Hong Kong for >30 years (*9*). Two of the case-patients were chefs, 1 of whom recalled having a minor puncture wound while cleaning grass carp ≈1 week before hospital admission. Another case involved a part-time fishmonger. Zoonotic *Streptococcus iniae* infection after handling raw fish, especially by persons with a puncture wound, was previously noted in Hong Kong (*13*,*14*). Thus, contact with raw fish may also be a transmission route for iGBS infection. The CHP introduced public health measures to enhance proper handling of raw fish and advised persons to thoroughly cook freshwater fish (*9*).

## Conclusions

We report a cluster of invasive GBS ST283 infections in nonpregnant adults in the late summer of 2021 and found the same ST in freshwater fish and environmental samples in wet markets of Hong Kong during that period. Because selling raw freshwater fish sashimi is prohibited locally, the main association of the upsurge was contact with or improper handling of freshwater fish, highlighting the zoonotic potential of GBS ST283 transmission through contact with freshwater fish.

Appendix 1 Sequence type (ST) 283 genomes (n = 303) from National Center for Biotechnology Database used for comparison with ST283 isolates from study of cluster of invasive group B *Streptococcus* infections caused by hypervirulent clone of *S. agalactiae* ST283, Hong Kong, China, 2021.

Appendix 2 Additional results from study of cluster of invasive group B streptococcal infections caused by hypervirulent clone of *Streptococcus agalactiae* sequence type 283, Hong Kong, China, 2021.
