# Supplementary material for: Invasive Group B Streptococcus Infections Caused by Hypervirulent Clone of S. agalactiae Sequence Type 283, Hong Kong, China, 2021
Source: Emerg Infect Dis. 2025 Jan;31(1):149–54. doi: 10.3201/eid3101.231627 (PMC11682793; doi:10.3201/eid3101.231627)
Supplement: Appendix 2 — Additional results from study of cluster of invasive group B streptococcal infections caused by hypervirulent clone of Streptococcus agalactiae sequence type 283, Hong Kong, China, 2021. [file 23-1627-Techapp-s2.pdf]

Article DOI: https://doi.org/10.3201/eid3101.231627

EID cannot ensure accessibility for supplementary materials supplied by authors. Readers who have difficulty accessing supplementary content should contact the authors for assistance.

Invasive Group B *Streptococcus* Infections Caused by Hypervirulent Clone of *S. agalactiae* Sequence Type 283, Hong Kong, 2021

Appendix 2

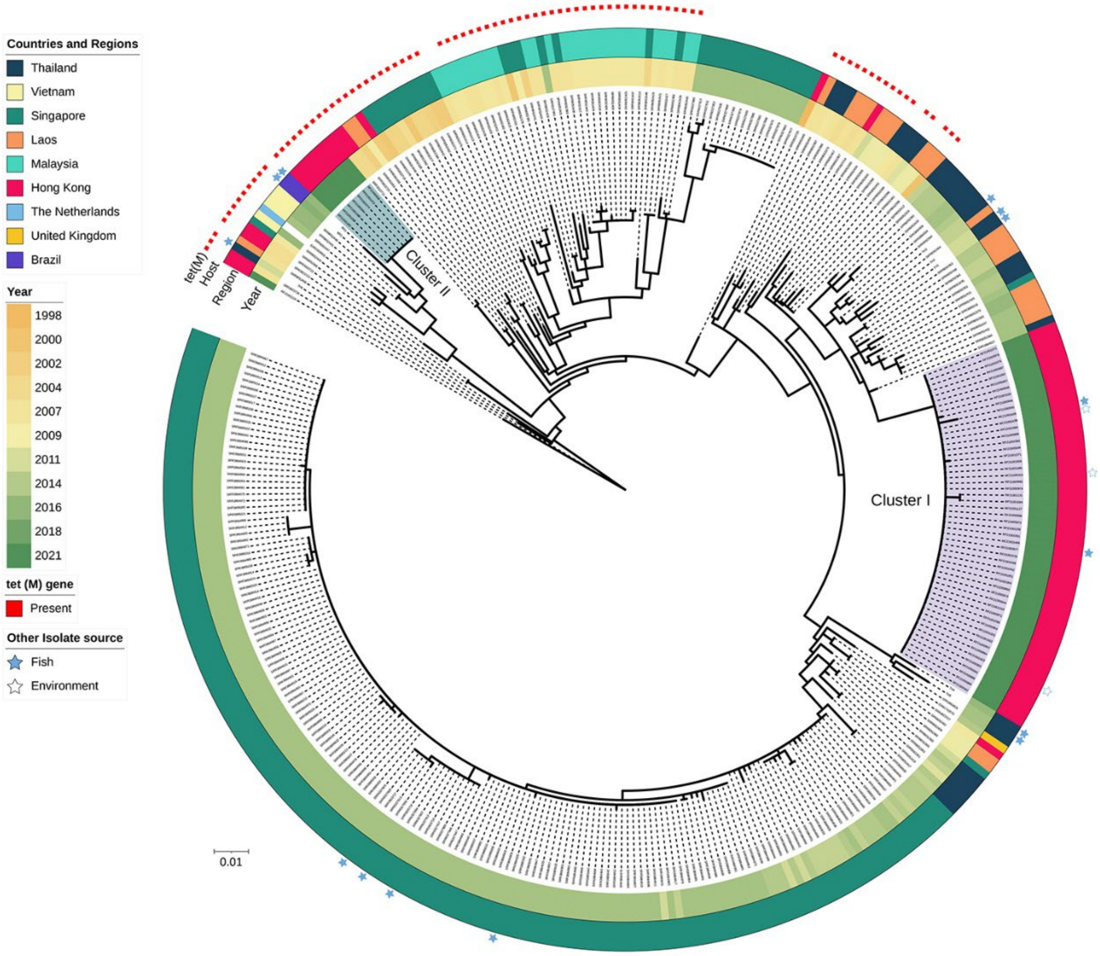

Appendix 2 Figure. Phylogeny of iGBS ST283 in Hong Kong Special Administrative Region, People's Republic of China from our study and global ST283 sequence data. GBS ST283 strains from our study were compared with ST283 sequence data across the globe (n = 303). The country/ region, and year of

isolation were noted. Isolates from fish (solid blue star) and the environment (hollow blue star) were marked under Host, while the remaining strains with no marking were clinical isolates. Presence of *tetM* gene (red square) was noted. Our two isolate clusters were highlighted (Cluster I and Cluster II with shadows of purple and blue shades respectively). Cluster I is the predominant group with the absence of *tetM*, and cluster II is the minor group that carries *tetM* gene.
